# Migalastat HCl Reduces Globotriaosylsphingosine (Lyso-Gb_3_) in Fabry Transgenic Mice and in the Plasma of Fabry Patients

**DOI:** 10.1371/journal.pone.0057631

**Published:** 2013-03-05

**Authors:** Brandy Young-Gqamana, Nastry Brignol, Hui-Hwa Chang, Richie Khanna, Rebecca Soska, Maria Fuller, Sheela A. Sitaraman, Dominique P. Germain, Roberto Giugliani, Derralynn A. Hughes, Atul Mehta, Kathy Nicholls, Pol Boudes, David J. Lockhart, Kenneth J. Valenzano, Elfrida R. Benjamin

**Affiliations:** 1 University of Cape Town, Institute of Infectious Disease and Molecular Medicine, Division of Medical Biochemistry, Cape Town, South Africa; 2 Amicus Therapeutics, Cranbury, New Jersey, United States of America; 3 Lysosomal Diseases Research Unit, South Australia Pathology at Women’s and Children’s Hospital, Adelaide, South Australia, Australia; 4 Division of Medical Genetics, University of Versailles – Saint Quentin en Yvelines, Hôpital Raymond Poincaré (Assistance Publique - Hôpitaux de Paris), Garches, France; 5 Medical Genetics Service, Clinic Hospital of Porto Alegre Federal University of Rio Grande do Sul, Porto Alegre, Brazil; 6 Lysosomal Storage Disorders Unit, Department of Academic Haematology, Royal Free Hospital and University College Medical School, London, United Kingdom; 7 Department of Nephrology, Royal Melbourne Hospital, Parkville, Victoria, Australia; Baylor Research Institute, United States of America

## Abstract

Fabry disease (FD) results from mutations in the gene (*GLA*) that encodes the lysosomal enzyme α-galactosidase A (α-Gal A), and involves pathological accumulation of globotriaosylceramide (GL-3) and globotriaosylsphingosine (lyso-Gb_3_). Migalastat hydrochloride (GR181413A) is a pharmacological chaperone that selectively binds, stabilizes, and increases cellular levels of α-Gal A. Oral administration of migalastat HCl reduces tissue GL-3 in Fabry transgenic mice, and in urine and kidneys of some FD patients. A liquid chromatography-tandem mass spectrometry method was developed to measure lyso-Gb_3_ in mouse tissues and human plasma. Oral administration of migalastat HCl to transgenic mice reduced elevated lyso-Gb_3_ levels up to 64%, 59%, and 81% in kidney, heart, and skin, respectively, generally equal to or greater than observed for GL-3. Furthermore, baseline plasma lyso-Gb_3_ levels were markedly elevated in six male FD patients enrolled in Phase 2 studies. Oral administration of migalastat HCl (150 mg QOD) reduced urine GL-3 and plasma lyso-Gb_3_ in three subjects (range: 15% to 46% within 48 weeks of treatment). In contrast, three showed no reductions in either substrate. These results suggest that measurement of tissue and/or plasma lyso-Gb_3_ is feasible and may be warranted in future studies of migalastat HCl or other new potential therapies for FD.

## Introduction

Fabry disease (FD, OMIM # 301500) is an X-linked lysosomal storage disorder caused by mutations in the gene (*GLA*; Gene/Locus MIM # 300644, Ref Seq NM_000169.2) that encodes the lysosomal hydrolase α-galactosidase A (α-Gal A, EC 3.2.1.22) [1]. Mutations in *GLA* that are associated with FD lead to reduced cellular α-Gal A activity [1]. Deficiency of α-Gal A results in accumulation of neutral glycosphingolipids with terminal α-galactosyl residues, primarily globotriaosylceramide (GL-3, Gb_3_, ceramide trihexoside), in the plasma, and in lysosomal and non-lysosomal compartments of cells of the blood vessels, skin, heart, kidney, brain, and other tissues and organs throughout the body [1], [2], [3], [4].

FD clinical manifestations include progressive renal failure, cardiac disease, cerebrovascular disease, small-fiber peripheral neuropathy, and skin lesions, among other abnormalities [1], [5]. The clinical presentation of FD spans a broad spectrum of severity, and roughly correlates with residual α-Gal A activity [1]. Males with FD who have little or no detectable α-Gal A activity are commonly referred to as “classic” Fabry patients and are most severely affected. Female Fabry patients may be mildly symptomatic or as severely affected as classic males [6]. Many individuals with FD present with a “later-onset” form, and generally have higher residual α-Gal A activity than “classic” patients [7].

Recently, the deacylated GL-3 analogue, globotriaosylsphingosine (known as lyso-Gb_3_), was found to be markedly increased in the plasma of “classic” male Fabry patients relative to that of normal individuals [8]. The relative excess of plasma lyso-Gb_3_ exceeded that of plasma GL-3 by more than an order of magnitude [8], [9], [10]. In symptomatic Fabry females, plasma lyso-Gb_3_ levels were clearly higher, while plasma GL-3 concentrations were in the normal range [8], [9], [10]. High levels of plasma lyso-Gb_3_ correlated with increased risk for cerebrovascular disease or left ventricular hypertrophy in FD males or females, respectively [9]. Greater life-time exposure to plasma lyso-Gb_3_ was found to correlate with disease severity in male and female patients with FD [9]. These observations suggest that plasma lyso-Gb_3_ is an important indicator of FD and warrants further evaluation as a marker of FD clinical severity and progression.

Currently, the only treatment available for Fabry patients is enzyme replacement therapy (ERT), with two approved products: Fabrazyme® (agalsidase beta; Genzyme Corporation, Cambridge, MA) and Replagal® (agalsidase alfa; Shire Pharmaceuticals, Cambridge, MA), generally given as regular every-other-week infusions. In humans, ERT is generally well-tolerated, and in some patients leads to lower levels of plasma, urine, and microvascular endothelial GL-3, stabilized kidney function, and improved FD-related clinical symptoms [11], [12], [13], [14], [15], [16]. Recently, reduction of plasma lyso-Gb_3_ levels in Fabry patients in response to ERT has been demonstrated [8], [10], [17]. Plasma lyso-Gb_3_ reduction was significantly lower in Fabry males who developed neutralizing antibodies towards the infused enzyme compared to those who did not [18].

A new approach to the treatment of Fabry disease, and which may serve as an alternative to ERT for some patients, is small-molecule pharmacological chaperone (PC) therapy [19], [20], [21], [22], [23]. PCs selectively bind and stabilize some mutant forms of α-Gal A in the endoplasmic reticulum, facilitate proper protein folding and trafficking, and thereby increase lysosomal enzyme activity. An investigational, orally available small molecule PC, migalastat hydrochloride (1-deoxygalactonojirimycin HCl, AT1001, GR181413A) is in Phase 3 clinical studies to evaluate its safety and efficacy as a potential treatment for FD (see ClinicalTrials.gov: NCT00925301 and NCT01218659). In pre-clinical studies, oral administration of migalastat HCl reduced GL-3 levels in plasma and disease-relevant tissues of Fabry transgenic mice (hR301Q α-Gal A Tg/KO and TgM/KO mice) [24], [25]. Furthermore, in Phase 2 clinical studies, oral administration of migalastat HCl reduced GL-3 levels in urine and in kidneys of some Fabry patients [26]. To date, the effect of migalastat HCl on plasma or tissue levels of lyso-Gb_3_ has not been evaluated in pre-clinical or clinical studies.

In this study, we developed methods for the detection and quantification of lyso-Gb_3_ in mouse tissues and human plasma using liquid chromatography-tandem mass spectrometry (LC-MS/MS). These methods were used to analyze lyso-Gb_3_ levels in disease-relevant tissues of *GLA* deficient (*GLA* KO) and hR301Q α-Gal A Tg/KO mice at baseline, after intravenous administration of rhα-Gal A (agalsidase beta), or after oral administration of migalastat HCl. The effects of these drug treatments on tissue lyso-Gb_3_ levels were compared to their effects on tissue GL-3 levels determined from the same mice. Lastly, plasma lyso-Gb_3_ was analyzed in six male subjects with FD who were administered migalastat HCl in Phase 2 clinical studies (see ClinicalTrials.gov: NCT00283959 and NCT00283933) [26]. Again, the effect of migalastat HCl treatment on plasma lyso-Gb_3_ levels was compared to the effects on urine and plasma GL-3 in the same subjects. The results show that measurement of tissue or plasma lyso-Gb_3_ levels, in addition to GL-3 levels, in response to ERT or oral administration of migalastat HCl is feasible and may be warranted in future pre-clinical and clinical studies.

## Materials and Methods

### Materials

Globotriaosylceramide (GL-3), lactosylceramide, globotriaosylsphingosine (lyso-Gb_3_) and plant glucopsychosine were purchased from Matreya LLC (Pleasant Gap, PA). Migalastat HCl was synthesized by Cambridge Major Laboratories (Germantown, WI). Recombinant human α-Gal A (rhα-Gal A; agalsidase beta; Fabrazyme®) was purchased from Genzyme Corporation (Cambridge, MA). Analytical grade methanol, acetonitrile, dimethysulfoxide (DMSO), acetone, sodium acetate and formic acid were purchased from Thermo Fisher Scientific (Waltham, MA). Deionized water was generated using a Mili-Q UV Plus water purifying system from Millipore (Billerica, MA).

### Mice/breeding

Mice that express a mutant transgene of human α-Gal A (R301Q) on a *GLA* knock-out (KO) mixed background of C57BL/6 and B129Sve (hR301Q α-Gal A Tg/KO) and *GLA* deficient (*GLA* KO) mice were obtained from Dr. Robert Desnick (Mt. Sinai School of Medecine, New York, NY). Wild-type C57BL/6 mice were purchased from Taconic Farms (Germantown, NY).

### Oral Administration of Migalastat HCl to hR301Q α-Gal A Tg/KO Mice

Migalastat HCl was administered orally to mice *ad libitum* in drinking water as described previously [25]. Briefly, migalastat HCl dosing solutions were made fresh weekly, with appropriate concentrations determined based on the average daily water consumption of hR301Q α-Gal A Tg/KO mice (∼5 mL/day per mouse) (all doses represent the free-base equivalent of the salt form). At study completion, mice were euthanized with CO_2_. Whole blood was drawn into lithium heparin tubes from the inferior vena cava and plasma was collected by centrifuging blood at 2,700 g for 10 minutes at 4°C. Heart, kidney, brain, and skin (shaved and removed from the lower ventral side of the neck) were quickly removed, rinsed in cold phosphate-buffered saline (PBS), blotted dry, and stored on dry ice.

### Tissue Homogenate Preparation Procedure

Tissue homogenates were prepared by adding 16 µL of deionized water per mg of tissue (typically 15 to 25 mg). The mixture was homogenized with lysing matrix A/D on a FastPrep-24 homogenizer (MP Biomedicals, Solon, OH). Care was taken to ensure complete homogenization of the tissue sample.

### Determination of GL-3 in Plasma or Tissue Homogenate

GL-3 was extracted from plasma (human and mouse) or mouse tissue homogenate by solid phase extraction (SPE) and analyzed via LC-MS/MS as previously described [25], [26]. Final tissue GL-3 concentrations were reported normalized to tissue weight. GL-3 in human whole urine was determined and normalized to total phosphatidylcholine (PC) as previously described [26].

### Lyso-Gb_3_ Calibration Standard and Quality Control (QC) Sample Preparation

Stock solutions of lyso-Gb_3_ and the internal standard (IS) glucopsychosine were prepared by dissolving the powders in a solvent mixture of chloroform/methanol (2/1, v/v) at final concentrations of 1 mg/mL. Two different stock solutions were used to prepare calibration standard or quality control samples in two steps. In the first step, one stock solution of lyso-Gb_3_ was used to prepare calibration standard spiking solutions in DMSO. A separate stock solution of lyso-Gb_3_ was used to prepare quality control spiking solution in DMSO. In the second step, the lyso-Gb_3_ calibration standard or QC spiking solution was added to plasma or tissue homogenate (at a dilution of 99/1, v/v; plasma or tissue homogenate/spiking solution) to prepare 8 matrix calibration standard levels: 1, 2, 5, 10, 25, 50, 100, 250 ng/mL and up to 5 matrix QC levels: 2, 4, 40, 80, 160, 200. The matrices (normal control human or wild-type mouse plasma or tissue homogenate) were pre-screened for levels of interfering peaks to lyso-Gb_3_ or the IS. Blank matrices found to have low or undetectable levels of interfering lipids were used.

### Lyso-Gb_3_ Tissue or Plasma Sample Extraction Procedure

A 50 µL aliquot of the plasma or tissue homogenate was transferred to a 13 mL silanized glass tube and 25 µL of the internal standard (500 ng/mL glucopsychosine in DMSO) was added. The mixture was further diluted with 1 mL of methanol, vortexed briefly, then sonicated at room temperature for approximately 10 minutes. After the addition of 500 µL of 1 N HCl, the mixture was shaken on medium speed (setting = 5) on a multi-tube vortexer (VWR, Radnor, PA) for approximately 30 minutes and then centrifuged at 3,220 g for 5 minutes at room temperature. The supernatant was loaded onto a pre-conditioned Oasis MCX 3 cc, 60 mg sorbent solid phase extraction cartridge (Waters, Milford, MA) and lyso-Gb_3_ extracted as previously described [27].

### Analytical Run Composition

All analytical runs were populated with double blanks (normal control human or wild-type mouse plasma or tissue homogenate that was run to check for interfering chromatographic peaks), blanks (normal control human or wild-type mouse plasma or tissue homogenate fortified with IS and used to check the IS response), two sets of calibration standards (one at the beginning of the run, and the other at the end), and QC samples in triplicate randomly placed within the runs.

### HPLC Instrumental Conditions

Chromatographic separation of lyso-Gb_3_ and IS was conducted using a liquid chromatography (LC) system that consisted of an HTc autosampler coupled with two LC-20AD pumps from Shimadzu (Columbia, MD). The chromatographic separation was performed at room temperature under a gradient elution profile using a Halo HILIC 2.7 µm, 75×4.6 mm silica analytical column from MAC MOD (Chadds Ford, PA). The following binary mobile phase system was used: A: 5 mM ammonium formate and 0.5% formic acid in acetonitrile/H_2_O (5/95, v/v), and B: 5 mM ammonium formate and 0.5% formic acid in acetonitrile/H_2_O (95/5, v/v). The following gradient profile was used to elute lyso-Gb_3_ and IS from the analytical column: 0.00 to 1.0-min/100% B, 1.01 to 4.00-min/100% to 70% B, 4.01 to 6.00-min/70% B, 6.01 to 7.50-min/60% B, 7.51 to 11∶00-min/100% B, and 11∶01-min/stop.

### MS/MS Instrumental Conditions

Tandem mass (MS/MS) spectrometry detection of lyso-Gb_3_ and IS was performed using a 4000QTrap mass spectrometer (Applied Biosystems, Foster City, CA). All optimization was performed via FIA with the above mentioned Shimadzu LC system (see HPLC instrumental conditions) at a flow rate of 0.5 mL/minute and a 10 µL injection. Positive ion electrospray ionization (ESI+) was used with the following conditions: an ion spray voltage of +5500V, a source temperature of 500°C, a curtain gas flow of 30 psi, a Gas1 flow of 20 psi, a Gas2 flow of 60 psi, a de-clustering potential of +141V for lyso-Gb_3_ and +56V for the IS. Nitrogen was used as the collision gas with a pressure of 6.00 mTorr. The collision energy was set at +53V for lyso-Gb_3_ and +29V for the IS. Quantitative MS/MS data were collected using selected reaction monitoring (SRM) scan mode with precursor ion to product ion transitions of *m/z* 787 to *m/z* 282 for lyso-Gb_3_ and *m/z* 460 to *m/z* 280 for the IS. These transitions represent the neutral loss of hexose from these molecules. The total run time was 11 minutes. All MS/MS data were acquired and analyzed using Analyst version 1.4.2 (Applied Biosystems).

### Determination of Lyso-Gb_3_ in Plasma and Tissues Samples

A linear calibration curve with weighting factor 1/x was generated by plotting the ratio of the peak area of lyso-Gb_3_ to that of the IS versus increasing standard actual concentrations of lyso-Gb_3_ in plasma or tissue homogenate. The standard curve was used to quantify lyso-Gb_3_ levels in the study samples.

### Extraction Efficiency of Lyso-Gb_3_ from Plasma and Tissue Homogenates

The extraction efficiency (also referred to as “recovery”) of lyso-Gb_3_ from biological matrices across the dynamic range of the current method was assessed at three concentration levels in plasma or tissue homogenates. The mean % recovery of lyso-Gb_3_ was defined as the ratio of the mean peak areas determined from sample extracts to those determined from blank matrix extracts that were spiked with lyso-Gb_3_ at similar concentrations after extraction.

### Data Analysis

Percent bias (% Bias) was defined as 100 times the difference between the mean found concentration and the actual concentration divided by the actual concentration [% Bias = 100×[(mean found concentration – actual concentration)/actual concentration]. Precision or percent coefficient of variation (% CV) was defined as 100 times the standard deviation divided by the mean found concentration [% CV = 100×(standard deviation/mean found concentration)].

Determinations of statistical significance were conducted using GraphPad Prism, version 5 (San Diego, CA). In the mouse studies, percent reduction (or percent change) refers to the percent of the mean difference from untreated (or control), and was calculated using Excel 2003 (Microsoft, Redmond, WA) as follows: [(mean untreated – treated) ÷ mean untreated] * 100. In the plasma samples from male FD patients, percent reduction (or percent change) refers to the percent of the difference from baseline, and was calculated using Excel 2003 as follows: [(baseline – treated) ÷ baseline] * 100.

### Ethics Statement

All animal experiments including animal husbandry were conducted according to protocols approved by the Rutgers University Animal Care and Facilities Use Committee. Patients signed informed consent to future use of their samples for research related to FD. These plasma samples were obtained during two open-label, Phase 2 clinical studies (see ClinicalTrials.gov: NCT00283959 and NCT00283933), which received Ethical Committee/Institutional Review Board (IRB) approval and were conducted according to accepted standards of Good Clinical Practice (ICH-GCP) and in agreement with the Declaration of Helsinki [26].

## Results

### Chromatography and Calibration Curves

**Figure 1:**
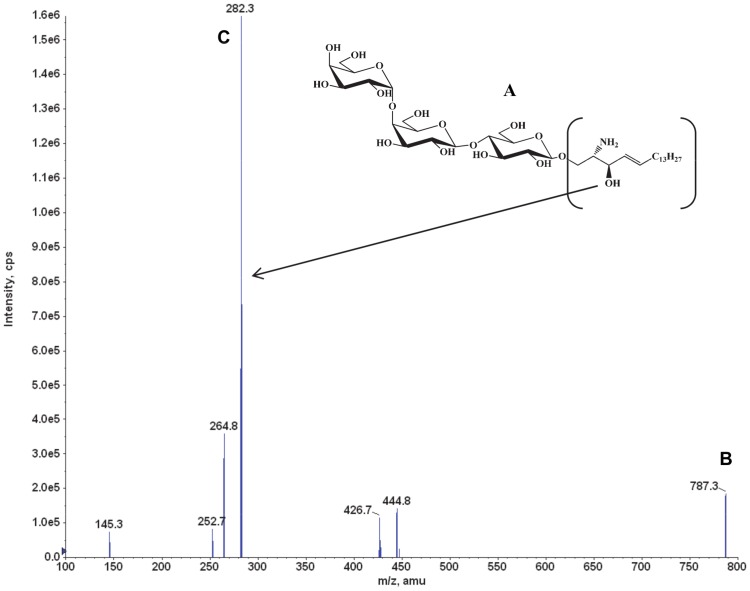
Lyso-Gb_3_: structure, MS, and product ion spectra. The lyso-Gb_3_ molecule (**A**) was detected and confirmed in positive electrospray ionization mode (ESI^+^), where (**B**) shows the lyso-Gb_3_ [M+H]^+^ ion at *m/z* of 787, and (**C**) shows the most intense product ion at *m/z* 282. The data were collected in product ion scan mode.

The base peak [M+H]^+^ at m/z = 787 was consistent with the lyso-Gb_3_ molecular weight of 787 (**Fig. 1A,B**). The product ion spectrum of m/z = 787 exhibited a major product ion at m/z = 282 (**Fig. 1C**). Thus, the SRM transition m/z = 787 → m/z = 282 was used to quantify lyso-Gb_3_ in plasma and tissue homogenates.

**Figure 2:**
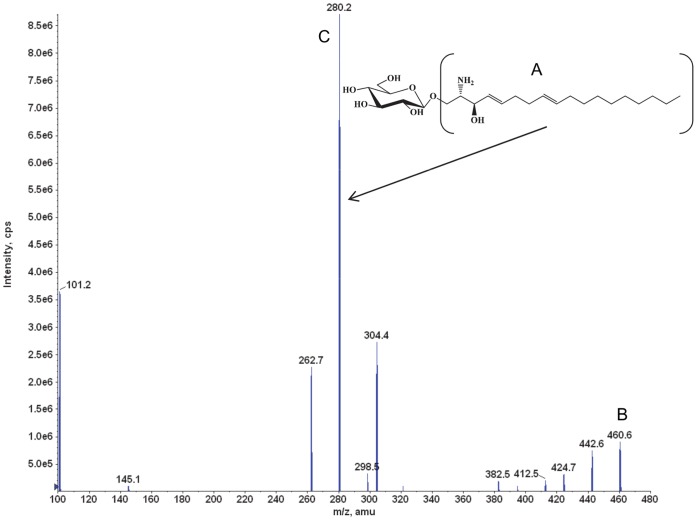
Glucopsychosine (IS): structure, MS, and product ion spectra. The glucopsychosine molecule (**A**) was detected and confirmed in positive electrospray ionization mode (ESI^+^), where (**B**) shows the glucopsychosine [M+H]^+^ ion at *m/z* of 460, and (**C**) shows the most intense product ion at *m/z* 280. The data were collected in product ion scan mode.

The base peak [M+H]^+^ at m/z = 460 was consistent with the glucopsychosine molecular weight of 460 (**Fig. 2A,B**). The product ion spectrum of m/z = 460 exhibited a major product ion at m/z = 280 (**Fig. 2C**). Therefore, the SRM transition m/z = 460 → m/z = 280 was used to quantify glucopsychosine in plasma and tissue homogenates.

**Figure 3:**
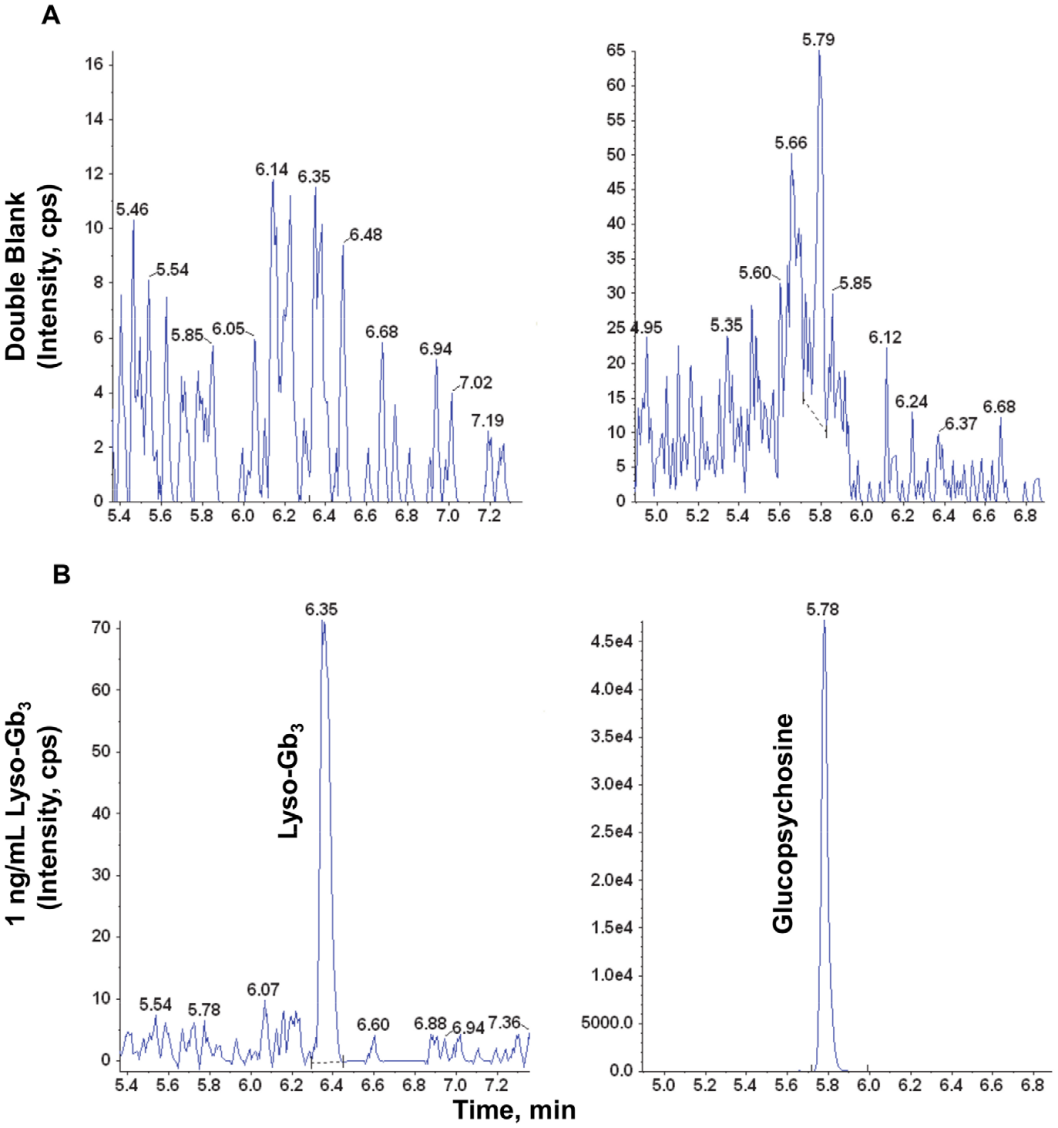
Representative LC-MS/MS chromatograms of lyso-Gb_3_ and glucopsychosine extracted from human plasma. (**A**) Blank human plasma without spiked lyso-Gb_3_ or glucopsychosine; (**B**) lyso-Gb_3_ calibration standard at LLOQ = 1 ng/mL; glucopsychosine (IS) in control human plasma.

The retention times for lyso-Gb_3_ and the glucopsychosine IS were approximately 6.3 minutes and 5.7 minutes, respectively, with an 11-minute run time (Figure 3). There were interfering peaks due to the presence of endogenous lyso-Gb_3_ and glucopsychosine in both normal control plasma and tissue homogenate samples. These interfering peaks were generally ≤25% of the lyso-Gb_3_ peak at the LLOQ (1 ng/mL) and ≤1% of the glucopsychosine peak at the concentration of 500 ng/mL used in this assay.

The linear regression y = ax+b with a weighting factor of 1/x was determined to best represent the relationship of the lyso-Gb_3_ concentration in plasma or tissue homogenates and the detector response (defined as the peak area ratio of lyso-Gb_3_ to the IS). Lyso-Gb_3_ levels from unknown samples were determined based on this calibration curve. The overall coefficient of determination r^2^ of the calibration curves in all matrices was ≥0.988.

### Assay Bias and Precision

QC samples were used to assess intra- and inter-assay bias and precision of the LC-MS/MS assay. QC samples were prepared in normal control human or wild-type mouse plasma or tissue homogenates at the LLOQ, low, middle, and high concentration range of the calibration curve, and were analyzed on at least two separate days. The QC samples were populated in the beginning, middle, and end of the analytical run to account for sample stability and possible instrumental drift. An intra-assay bias criterion was set, whereby the analytical run was accepted if 2/3 of the QC samples were within ±20% (±25% for the LLOQ) of the actual lyso-Gb_3_ concentration.

The intra-assay bias in the four mouse matrices ranged from −5.70% to 15.6% of actual values (data not shown). The intra-assay precision across all matrices was ≤16.6% (22.9% at the LLOQ) (data not shown). The inter-assay bias across the four mouse matrices ranged from −5.70% to 15.6% of actual values (**Table 1**). The inter-assay precision across the four matrices was ≤15.8% (**Table 1**). Inter- and intra-assay bias and precision were also assessed in quality control samples prepared in normal control human plasma, as described above. In summary, the intra-assay bias ranged from −12.8 to 15.6% of actual values (data not shown). The intra-assay precision ranged from 2.52 to 19.8% (data not shown). The inter-assay bias in human plasma ranged from −6.19 to 3.60% of actual values (**Table 2**). The inter-assay precision ranged from 9.25 to 16.4% (**Table 2**).

**Table 1:** Inter-assay % Bias and precision (% CV) of lyso-Gb_3_ quality control mouse tissue samples.

QC (Actual Conc.)		Kidney	Skin	Heart	Plasma
Low_1_(2.00 ng/mL)	**Mean ± SD**	2.16±0.306	2.02±0.233	2.12±0.122	2.15±0.210
	**% CV**	14.2	11.5	5.77	9.78
	**% Bias**	7.89	1.00	6.17	7.36
Low_2_(4.00 ng/mL)	**Mean ± SD**	4.10±0.327	4.18±0.517	4.23±0.413	4.09±0.616
	**% CV**	8.0	12.4	9.78	15.1
	**% Bias**	2.50	4.43	5.67	2.25
Mid(80.0 ng/mL)	**Mean ± SD**	84.9±8.43	82.3±5.51	92.5±3.91	79.8±12.6
	**% CV**	9.93	6.70	4.23	15.8
	**% Bias**	6.08	2.88	15.6	−0.250
High(160 ng/mL)	**Mean ± SD**	162±19.7	167±13.9	181±27.2	151±11.8
	**% CV**	12.2	8.34	15.0	7.83
	**% Bias**	1.32	4.50	13.2	−5.70

Quality control samples were prepared in wild-type mouse plasma or tissue homogenates at four concentration levels and analyzed on three separate days. Six to 10 replicates at each concentration level were used for inter-assay determination. Conc., concentration; SD, Standard Deviation.

**Table 2:** Inter-assay % Bias and precision (% CV) of lyso-Gb_3_ quality control human plasma samples.

QC (actual conc.)	Low_1_ (2.00)	Low_2_ (4.00)	Mid_1_ (40.0)	Mid_2_ (80.0)	High (200)
**Mean ± SD (ng/mL)**	2.07±0.249	3.75±0.617	41.2±5.20	75.0±6.94	200±19.7
**% CV**	12.0	16.4	12.6	9.25	9.84
**% Bias**	3.60	−6.16	2.95	−6.19	0.15

Quality control samples were prepared in normal human plasma at five concentration levels and analyzed on four separate days. Twenty replicates at each concentration level were used for inter-assay determination. Conc., concentration (ng/mL); SD, Standard Deviation.

### Recovery of Lyso-Gb_3_ from Plasma and Tissue Homogenates

The mean % recovery of lyso-Gb_3_ from human plasma ranged from 76% to 104% at the three concentration levels used in this experiment (**Table 3**). The mean % recovery in mouse plasma or tissue homogenates was greater than or equal to 65% across the actual concentrations of lyso-Gb_3_ assessed in the experiment (**Table 3**).

**Table 3:** Recovery of lyso-Gb_3_ from plasma and tissue homogenates.

	% Recovery (Mean)				
	Human	Mouse			
Actual Conc.(ng/mL)	Plasma	Plasma	Skin	Heart	Kidney
**2**	76	80	70	88	76
**10**	94	76	64	66	71
**160**	104	87	69	86	77

Recovery samples were prepared in normal human plasma, and wild-type mouse tissue homogenates or plasma at three concentration levels and analyzed in quintuplicates (n = 5). % recovery (mean) = 100×(mean peak area of extracted lyso-Gb_3_/mean peak area of unextracted lyso-Gb_3_).

### Determination of Lyso-Gb_3_ in Normal and Fabry Mouse Tissues

**Figure 4:**
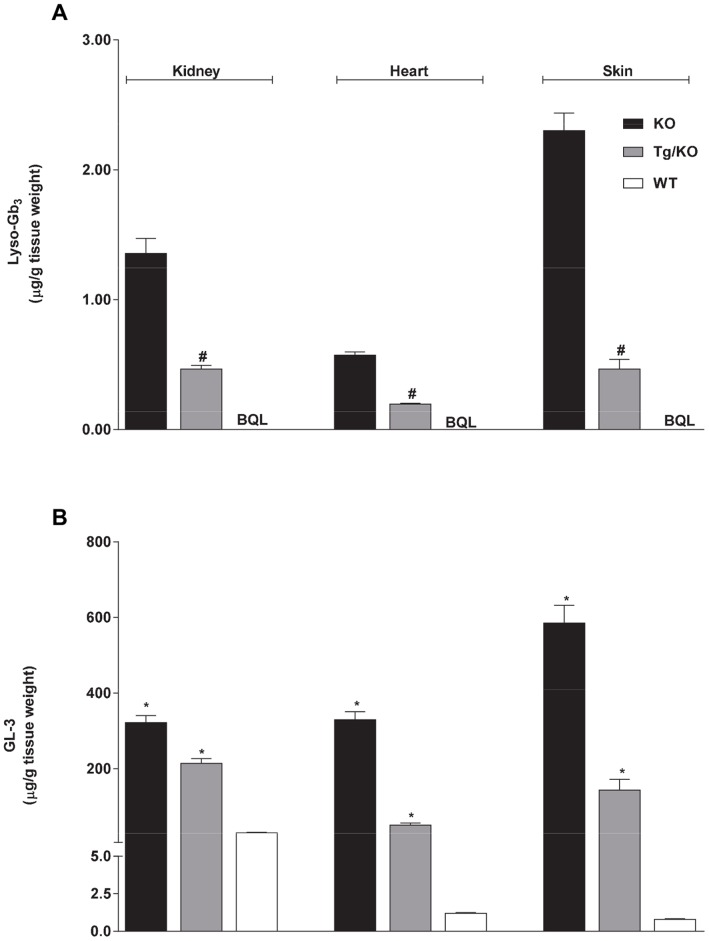
Baseline levels of lyso-Gb_3_ and GL-3 in normal and Fabry mouse tissues. Baseline levels of (**A**) lyso-Gb_3_ and (**B**) GL-3 were measured in kidney, heart, and skin tissues of twelve-week-old male wild-type (C57BL/6; WT), hR301Q α-Gal A Tg/KO (Tg/KO), and *GLA* KO (KO) mice. *p<0.05 compared to WT, t-test; #p<0.05 compared to KO, t-test; WT contained non-detectable levels of lyso-Gb_3_. BQL = Below Quantitation Limit <0.034 ug/g tissue weight; the lyso-Gb_3_ and GL-3 data represent the mean ± SEM of 5–10 mice/group.

An α-Gal A gene knock-out (*GLA* KO) mouse model of FD that shows significant accumulation of GL-3 in multiple tissues including skin, heart, and kidney has been described [25], [28], [29], [30]. In addition, a new mouse model of Fabry disease that expresses a human R301Q *GLA* transgene transcriptionally regulated by the human *GLA* promoter on a *GLA* KO background (hR301Q α-Gal A Tg/KO) has also been shown to accumulate GL-3 in Fabry disease-relevant tissues [25]. Recently, the *GLA* KO mice have been shown to accumulate lyso-Gb_3_ in multiple tissues, as measured by o-phtaldialdehyde (OPA)-derivitization of lyso-Gb_3_ followed by HPLC-fluorescence detection (HPLC-FD) [8], [31]. However, lyso-Gb_3_ levels have not yet been characterized in the hR301Q α-Gal A Tg/KO mice. Thus, to assess whether tissue lyso-Gb_3_ accumulation in *GLA* KO mice can be reproduced using our LC-MS/MS assay, and to extend the characterization of the hR301Q α-Gal A Tg/KO mouse model, the baseline levels of lyso-Gb_3_ in heart, kidney, and skin tissues were measured in 12-week old male wild-type (C57BL/6), hR301Q α-Gal A Tg/KO, and *GLA* KO mice by LC-MS/MS (**Fig. 4A**). GL-3 levels were also measured by LC-MS/MS in the same tissue samples for comparison (**Fig. 4B**).

Lyso-Gb_3_ levels were below the lower limit of quantification (BQL <1 ng/mL) in wild-type mouse tissues, but were markedly elevated in hR301Q α-Gal A Tg/KO and *GLA* KO mice. *GLA* KO mice showed significantly higher tissue levels of lyso-Gb_3_ (2.9 to 4.9-fold) compared to those found in hR301Q α-Gal A Tg/KO mouse tissues. The trend seen for lyso-Gb_3_ levels (*i.e., GLA* KO mice>hR301Q α-Gal A Tg/KO mice>wild-type mice) was similar for GL-3 levels determined in the same tissues of these transgenic and wild-type mice.

### Administration of rhα-Gal A Decreases lyso-Gb_3_ and GL-3 Levels in *GLA* KO Mice

**Figure 5:**
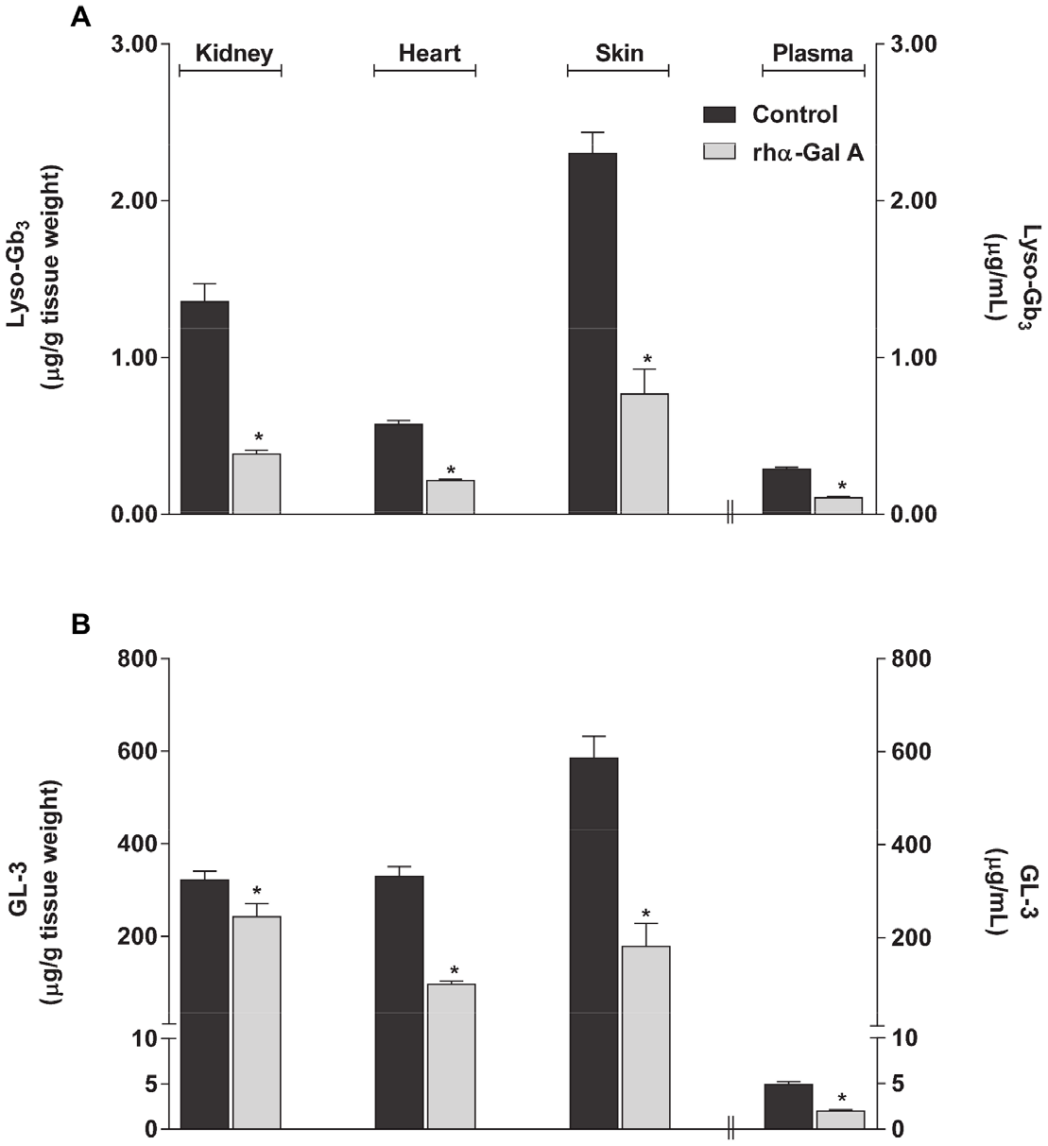
Lyso-Gb_3_ and GL-3 reductions in *GLA* KO mice administered rhα-Gal A. Twelve-week old male *GLA* KO mice were used as control or administered 1 mg/kg rhα-Gal A via bolus tail vein injection. Kidney, heart, skin, and plasma were harvested 7 days post-administration for the determination of (**A**) lyso-Gb_3_ and (**B**) GL-3 levels. The lyso-Gb_3_ and GL-3 data represent the mean ± SEM of 5 mice/group. *p<0.05 compared to untreated; t-test.

Intravenous administration of rhα-Gal A (agalsidase beta, 1 mg/kg body weight) to *GLA* KO mice has been shown to result in both decreased lyso-Gb_3_ and GL-3 levels as measured by non-LC-MS/MS methods (*e.g.*, HPLC-FD of OPA-derivitized lyso-Gb_3_ and high performance thin layer chromatography with immunostaining and luminescent imaging of GL-3) [31]. In that study, the observed relative decrease in kidney lyso-Gb_3_ was greater than the decrease in kidney GL-3. Thus, to assess whether a similar pattern of decrease can be reproduced using LC-MS/MS, lyso-Gb_3_ and GL-3 levels were determined in heart, kidney, skin, and plasma of *GLA* KO mice seven days after a single intravenous administration of rhα-Gal A (1 mg/kg agalsidase beta; **Fig. 5**). Lyso-Gb_3_ and GL-3 levels in all tissues and plasma were significantly (p<0.05) lower in the rhα-Gal A-treated animals compared to untreated animals. The reductions in lyso-Gb_3_ levels (mean ± std. err.) were −72±2%, −63±2%, −67±7%, and −63±3% in kidney, heart, skin and plasma, respectively. The reductions in GL-3 levels were −25±9%, −71±2%, −70±8%, and −59±3% in kidney, heart, skin, and plasma, respectively. Thus, consistent with previous observations, rhα-Gal A administered to *GLA* KO mice in this study generally resulted in similar reductions of lyso-Gb_3_ and GL-3 levels in most FD-relevant tissues and plasma, except in kidney where the reduction in lyso-Gb_3_ (−72%) was substantially greater than that seen for GL-3 (−25%).

### Administration of Migalastat HCl Decreases Tissue Lyso-Gb_3_ Levels in hR301Q α-Gal A Tg/KO Mice

Migalastat HCl is a pharmacological chaperone that can selectively bind, stabilize, and increase cellular levels of α-Gal A [20], [23], [32], [33], [34], [35]. Recently, oral administration of migalastat HCl was shown to reduce GL-3 levels in tissues of hR301Q α-Gal A Tg/KO mice [25]. In that study, dose optimization revealed that an even greater reduction in GL-3 was achieved using less-frequent administration as compared to daily administration of migalastat HCl. Thus, in the current study, we assessed the effect of migalastat HCl on tissue lyso-Gb_3_ levels in these mice using our LC-MS/MS assay, and compared these results to the effect observed on tissue GL-3 as determined from the same tissue samples. To this end, hR301Q α-Gal A Tg/KO mice were administered migalastat HCl (100 mg/kg) *ad libitum* in drinking water either daily or less frequently (four consecutive days with drug followed by three consecutive days with drinking water only; *i.e.,* 4 on/3 off) for 28 days.

**Figure 6:**
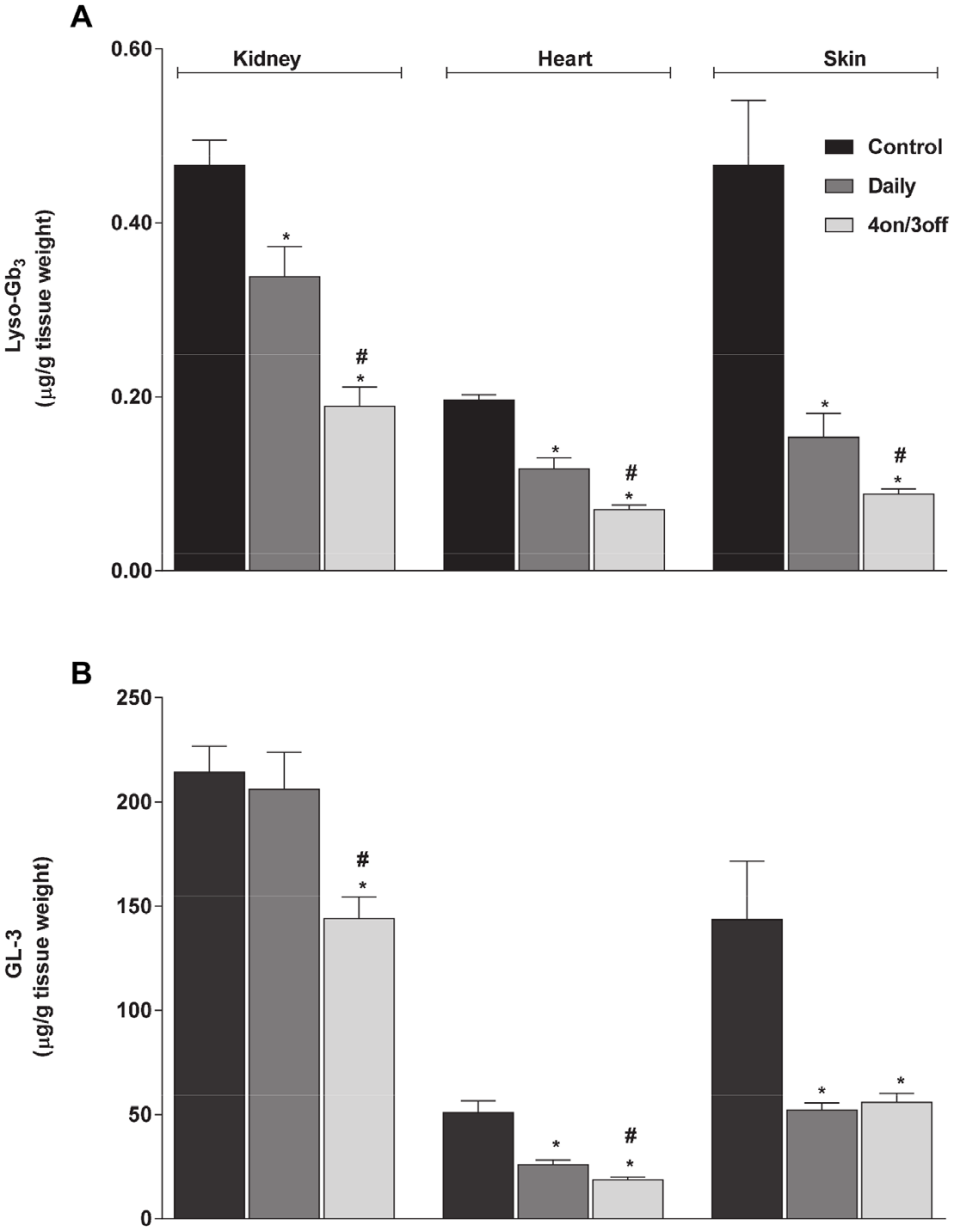
Lyso-Gb_3_ and GL-3 reduction in hR301Q α-Gal A Tg/KO mice administered migalastat HCl. Eight-week old male Fabry hR301Q α-Gal A Tg/KO mice were administered either water or migalastat HCl (100 mg/kg *ad libitum* in drinking water) daily or less frequently (4 on/3 off) for 28 days. Kidney, heart, and skin were subsequently harvested and analyzed for (**A**) lyso-Gb_3_ and (**B**) GL-3 levels. The lyso-Gb_3_ and GL-3 data represent the mean ± SEM of 10 mice/group. *p<0.05 compared to control; #p<0.05 compared to daily; t-test.

Daily and less-frequent administration of migalastat HCl to hR301Q α-Gal A Tg/KO mice resulted in significant reductions in lyso-Gb_3_ levels in kidney, heart, and skin (p<0.05 for all three tissues using either regimen) (**Fig. 6A**). Furthermore, significantly greater reductions in tissue lyso-Gb_3_ levels were seen with the “4 on/3 off” regimen compared to daily administration. Lyso-Gb_3_ reductions (mean ± std. err.) of −27±7%, −40±6%, and −67±6% were seen in kidney, heart, and skin, respectively, with daily migalastat HCl administration; reductions of −59±5%, −64±3%, and −81±1%, respectively, were seen with the less-frequent regimen. In general, similar effects were seen on GL-3 levels determined from the same tissues. GL-3 reductions of −4±8%, −49±4%, and −64±2% were seen in kidney, heart, and skin, respectively, with daily migalastat HCl administration; reductions of −33±5%, −63±3%, and −61±3%, respectively, were seen with the less-frequent regimen. In general, GL-3 reduction was greater with the less-frequent regimen (**Fig. 6B**), consistent with previous findings [25]. In addition, reduction of kidney lyso-Gb_3_ in hR301Q α-Gal A Tg/KO mice with either migalastat HCl regimen was substantially greater than the reductions in kidney GL-3, and was consistent with the greater reduction of lyso-Gb_3_ compared to GL-3 seen in *GLA* KO mouse kidney after administration of rhα-Gal A.

### The Effects of Migalastat HCl on lyso-Gb_3_ in Plasma of FD Patients

In two open-label, Phase 2 clinical studies (see ClinicalTrials.gov: NCT00283959 and NCT00283933) a total of nine male FD patients were administered 150 mg migalastat HCl orally every other day (QOD) for up to 48 weeks. Increases in peripheral blood mononuclear cell (PBMC), skin, and kidney α-Gal A activity of at least 50% were seen in 6 of 9 patients. GL-3 decreases were also seen in skin, urine, and kidney. In urine, all nine patients had elevated GL-3 levels prior to administration of migalastat HCl. In 5 of the 9 patients, urine GL-3 levels were lower by at least 20% at the last measured time point compared to baseline [26].

In the current study, the effects of treatment with migalastat HCl on plasma lyso-Gb_3_ levels were retrospectively assessed in a subset of male FD patients from these Phase 2 clinical studies. The effects on plasma lyso-Gb_3_ were compared to the effects on other potential biomarkers, such as plasma and urine GL-3, in the same patients. To this end, lyso-Gb_3_ and GL-3 levels were analyzed in plasma samples collected at baseline, as well as after 12, 24, and 48 weeks of migalastat HCl administration to six male FD patients during the Phase 2 studies. These six patients signed informed consent to future use of their samples for research related to FD. Furthermore, three of these FD patients had the *GLA* missense mutations p.N215S, p.P205T, and p.R301Q, and had shown increased PBMC α-Gal A activity and decreased urine GL-3 levels after migalastat HCl administration. The other three had *GLA* missense mutations, p.C94S, p.R112C and p.F295C, had shown increase in PBMC α-Gal A activity with no decrease in urine GL-3 levels after migalastat HCl administration at 150 mg QOD for up to 48 weeks [26].

**Figure 7:**
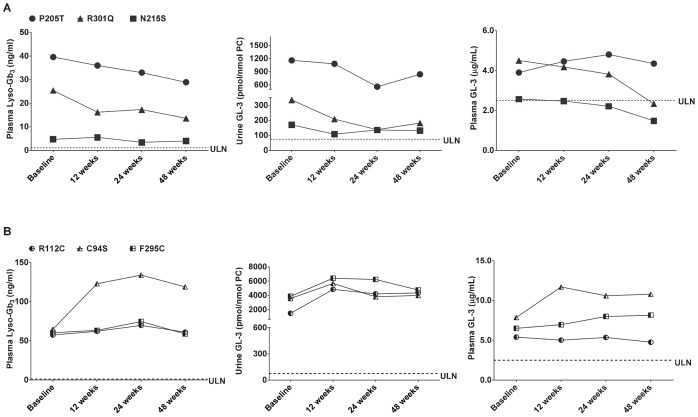
Plasma lyso-Gb_3_, as well as urine and plasma GL-3 levels in male FD patients after oral administration of migalastat HCl. (**A**) Male FD patients who showed urine GL-3 reductions after oral administration of migalastat HCl; (**B**) male FD patients who did not show urine GL-3 reductions after oral administration of migalastat HCl; ULN, upper limit of normal. The ULN was determined for urine GL-3, and the value is 74.6 pmol/nmol PC. The same acronym denotes the upper range of normal for plasma lyso-Gb_3_ (value is 1.12 ng/mL; [36]) and plasma GL-3 (value is 2.50 µg/mL).

The baseline plasma lyso-Gb_3_ levels of the six FD patients ranged from 4.70 to 64.9 ng/mL (**Fig. 7A, B** **; left panels)**. These levels were markedly elevated above normal (normal level <2.00 ng/mL in the current study (n = 6), consistent with the previously reported normal range of 0.12 to 1.12 ng/mL determined from 178 healthy control plasma samples; [36]). In the three FD patients with p.P205T, p.N215S, and p.R301Q mutations who had shown urine GL-3 changes ranging from −20% to −59% after 24 or 48 weeks on migalastat HCl (**Fig. 7A** **; middle panel)**, plasma lyso-Gb_3_ levels were also reduced with changes ranging from −15% to −46% at the same time points (**Fig. 7A** **; left panel**). In the three FD patients with p.C94S, p.R112C, and p.F295C mutations who had shown changes in urine GL-3 ranging from +7% to +192% after 24 or 48 weeks on drug (**Fig. 7B** **; middle panel**), plasma lyso-Gb_3_ levels showed changes of −2.6% to +106% (**Fig. 7B** **; left panel**).

In contrast to the marked baseline elevations seen for urine GL-3 and plasma lyso-Gb_3_, baseline plasma GL-3 levels were either moderately elevated or in the high-normal range in these six FD patients (range: 2.57 to 7.87 µg/mL compared to 1.15 to 2.50 µg/mL in 6 different normal plasma samples that were assessed in this study, and compared to 7 µg/mL upper limit of normal reported previously [37]). These results were consistent with the male FD patient plasma GL-3 ranges previously reported in multiple independent investigations [8], [9], [17]. In addition, plasma GL-3 changes after migalastat HCl administration (**Fig. 7A, B** **; right panels**) were not consistent with the changes seen in urine GL-3 or plasma lyso-Gb_3_ for some of the patients.

## Discussion

Lyso-Gb_3_, a deacylated analogue of GL-3, the primary substrate which accumulates in FD, has recently been shown to be elevated in the plasma of Fabry patients and is an important new indicator of FD [8]. In the current study, an LC-MS/MS method was developed that allows accurate and quantitative measurement of lyso-Gb_3_ in human plasma as well as in mouse skin, kidney, heart, and plasma. Preliminary assessment indicates that the method can also be applied to lyso-Gb_3_ quantification in mouse brain (data not shown).

Previous studies of lyso-Gb_3_ in mouse tissues have used non-LC-MS/MS methods with lower sensitivity (∼10 ng/mL LLOQ) that have required lengthy and complicated preparation procedures [8], [31]. These same methods were used to discover the presence of elevated lyso-Gb_3_ in the plasma of FD patients [8]. More recently, high sensitivity (∼ 2 ng/mL LLOQ in human plasma), rapid LC-MS/MS methods for measurement of lyso-Gb_3_ in human urine and plasma have been developed [27], [36], [38], [39]. One of these human plasma methods used a non-commercially available glycine derivative of lyso-Gb_3_ as an internal standard. Our method utilizes commercially-available plant glucopsychosine as the internal standard, the same internal standard used by Auray-Blais *et al.* for the measurement of lyso-Gb_3_ in human urine [27]; and by Boutin et al. for the measurement of lyso-Gb_3_ in human plasma [39]. A previously stated concern about the use of plant glucopsychosine as an internal standard was the risk of interference with the glucopsychosine measurement depending on patient nutrition status or consumption of a plant-rich diet [36], [38]. Thus, in our assays the concentration of the internal standard (500 ng/mL) that was used achieved peak area counts in a range where the contribution of endogenous lipid interference was limited to 1% or less of the internal standard response. In addition, the previously reported methods have not been evaluated in mouse tissues or plasma. Thus, the LC-MS/MS method we have developed has equivalent sensitivity, accuracy, precision, reliability, and simplicity, and works for a variety of sample and tissue types in mice and humans while not requiring access to specialty reagents.

We report the first LC-MS/MS analysis of lyso-Gb_3_ levels, with comparison to GL-3 levels, determined in multiple tissues from two different Fabry mouse models at baseline, after intravenous administration of rhα-Gal A, or after oral administration of the PC, migalastat HCl. The results reproduced previous findings of elevated baseline lyso-Gb_3_ in *GLA* KO mice as measured by non-LC-MS/MS methods [8], [31]. Interestingly, hR301Q α-Gal A Tg/KO mice showed intermediate levels of lyso-Gb_3_ and GL-3 in tissues compared to *GLA* KO and wild-type mice, perhaps due to the low but significant residual activity of the transgene [20], [25], [33], [40]. As shown previously [31], intravenous administration of rhα-Gal A to *GLA* KO mice reduced lyso-Gb_3_ and GL-3 levels to similar extents in multiple tissues, except kidney in which the lyso-Gb_3_ reduction was greater. Furthermore, similar reductions in lyso-Gb_3_ and GL-3 were seen across most tissues, except in kidney, of the transgenic mice after daily or less-frequent oral administration of migalastat HCl. In both Fabry mouse models, kidney GL-3, but not lyso-Gb_3_, consistently showed less reduction than other tissues in response to the different treatments and regimens. Less kidney GL-3 reduction in the transgenic mice after oral administration of miglastat HCl has been reported previously [25]; and may be due to differences in the turnover rates of the affected cell types in the various tissues, differences in the tissue concentrations and rates of clearance of the PC, or the possibility of GL-3 re-uptake into kidney from the urine [25]. In addition, it is interesting to note that our lyso-Gb_3_ and GL-3 results were from tissues of male mice. Unlike normal humans or FD patients [41], [42], mature male wild-type and *GLA* KO mice have markedly elevated urine and kidney GL-3 levels as compared to mature female wild-type and *GLA* KO mice [43]. This murine-specific sex difference is thought to be due to a testosterone-induced form of GL-3 that is secreted into the urine in multi-lamellar bodies [44], [45], [46], which appears to be inaccessible to exogenously administered rhα-Gal A [30]. Moreover, incomplete clearance of kidney GL-3 in male *GLA* KO mice has been reported in response to other forms of therapy [47], [48], [49], [50]. However, in one study of female TgM/KO mice expressing hR301Q under a β-actin promoter (a different hR301Q mouse model than used in the current studies), oral administration of migalastat HCl (3 mg/kg *ad libitum* in drinking water for 4 weeks) again led to an incomplete reduction (−46%) in kidney GL-3 levels [24], although GL-3 reduction in other tissues was not assessed. Thus, less GL-3 reduction in kidney as compared to other tissues of male Fabry mice reported here is consistent with previous results and may be due to a number of factors related to the kidney tissue, the drug treatments, and/or to the sex of the animals used in the studies. Importantly, the consistently greater reductions in kidney lyso-Gb_3_ as compared to GL-3 seen in the current studies suggest that kidney lyso-Gb_3_ may be a more sensitive indicator of increased kidney α-Gal A activity *in situ* in pre-clinical studies of male Fabry mice.

We also report the first analysis of lyso-Gb_3_ levels in plasma from male Fabry patients after oral administration of migalastat HCl, an investigational PC that is currently in Phase 3 studies for the treatment of FD (see ClinicalTrials.gov: NCT00925301 and NCT01218659). This retrospective analysis of samples obtained during Phase 2 clinical studies (see ClinicalTrials.gov: NCT00283959 and NCT00283933) [26] showed that the baseline plasma lyso-Gb_3_ levels in all six patients studied (6 to 83 nM) were markedly elevated above normal (normal <1.4 nM). The baseline plasma lyso-Gb_3_ range was lower, but overlapped with the previous reported plasma lyso-Gb_3_ range seen in males from a larger cohort of patients with “classic” FD (range: 51 to 489 nM; n = 37 [9]).

Three FD patients in the Phase 2 clinical studies had *GLA* missense mutations (p.N215S, p.P205T, and p.R301Q), and had shown increased PBMC α-Gal A activity and decreased urine GL-3 levels after migalastat HCl administration [26]. Based on the current study, these three FD patients also showed decreased plasma lyso-Gb_3_ levels after oral administration of migalastat HCl (150 mg QOD for up to 48 weeks). The other three FD patients had *GLA* mutations corresponding to p.C94S, p.R112C, and p.F295C, and had not shown increased PBMC α-Gal A activity or decreased urine GL-3 levels after migalastat HCl administration [26]. Based on the current study, these three FD patients also did not show decreased plasma lyso-Gb_3_ levels after oral administration of migalastat HCl. These results indicate that in this limited number of male FD patients, the change in plasma lyso-Gb_3_ was consistent with the change in urine GL-3 after oral administration of migalastat HCl. In contrast, plasma GL-3 changes from baseline were not consistent with the changes seen in urine GL-3 or plasma lyso-Gb_3_ for some of the patients. Furthermore, the baseline plasma GL-3 levels were not clearly elevated in all of the patients, consistent with high-normal plasma GL-3 ranges previously reported in male FD patients [8], [9], [10], [17].

In the three FD patients that showed plasma lyso-Gb_3_ reductions, the magnitudes of the changes (−15% to −47%) after 24 to 48 weeks of migalastat HCl administration were lower than those (*e.g*., −68%, n = 22) seen in male classic FD patients after 48 weeks of treatment with different ERT regimens [8], [10], [17]. However, in those previous studies, the baseline plasma lyso-Gb_3_ levels in the ERT-treated male patients were markedly greater (102 to 397 nM, ∼250 to 450 nM, and ∼75 nM respectively). It is possible that greater plasma lyso-Gb_3_ reductions may be dependent, in part, on greater elevation at baseline; further investigation is warranted.

For more than a decade, monitoring plasma or urine GL-3 levels as a diagnostic tool for FD and as a marker of treatment efficacy has been evaluated in numerous independent investigations [51], [52]. Urine GL-3 was more consistently elevated as compared to plasma GL-3, particularly in females with FD. However, for some females and a few exceptional males with certain ‘later-onset’ disease-associated mutations (*e.g.*, N215S and M296I), urine GL-3 was in the normal range, limiting its diagnostic value for such patients [53]. More recently, plasma lyso-Gb_3_ has shown greater sensitivity than plasma GL-3 as an indicator of FD in females [9]. However, a direct and thorough comparison of the sensitivity of plasma lyso-Gb_3_ to that of urine GL-3 is not yet available. The current study provides an initial retrospective assessment of plasma lyso-Gb_3_ and urine GL-3 levels at baseline and in response to oral administration of migalastat HCl in samples from the same FD patients. In the small number of male FD patients tested, plasma lyso-Gb_3_ and urine GL-3 showed comparable results. Future clinical investigation to extend the comparison of urine GL-3 and plasma lyso-Gb_3_ to larger FD patient cohorts, preferably matched for age, sex, disease severity, and *GLA* genotype is warranted.

In conclusion, we have developed a sensitive and robust method for the detection of lyso-Gb_3_ levels in mouse tissues and human plasma using LC-MS/MS. We have found that lyso-Gb_3_ is elevated in disease-relevant tissues of a transgenic mouse model of Fabry disease. We have shown significant reductions in the transgenic mouse tissue lyso-Gb_3_ levels in response to daily or less-frequent oral administration of migalastat HCl. The tissue lyso-Gb_3_ reductions were generally equal to or greater than those of GL-3 determined from the same mice. The LC-MS/MS method was also used to retrospectively analyze plasma lyso-Gb_3_ levels in six male subjects orally administered migalastat HCl (150 mg QOD) in Phase 2 clinical studies [26]. Importantly, the results show for the first time that migalastat HCl can lower plasma lyso-Gb_3_ levels in some FD patients treated with this PC. Thus, the LC-MS/MS method developed here represents a single approach that enables accurate measurement of lyso-Gb_3_ in pre-clinical and clinical studies of investigational new therapies for the treatment of FD, as well as in new studies of currently available ERTs. As migalastat HCl is in Phase 3 clinical studies to further assess its safety and efficacy for the treatment of FD, the results from the current studies suggest that monitoring of lyso-Gb_3_, in addition to other assessments, is feasible and warranted in future prospective or retrospective studies.
